# Social Behaviours and Networks of Vervet Monkeys Are Influenced by Gastrointestinal Parasites

**DOI:** 10.1371/journal.pone.0161113

**Published:** 2016-08-31

**Authors:** Colin A. Chapman, Sagan Friant, Kathleen Godfrey, Cynthia Liu, Dipto Sakar, Valérie A. M. Schoof, Raja Sengupta, Dennis Twinomugisha, Kim Valenta, Tony L. Goldberg

**Affiliations:** 1 McGill School of Environment and Department of Anthropology, McGill University, Montreal, Quebec, Canada, H3A 2T7; 2 Wildlife Conservation Society, Bronx, New York, 10460, United States of America; 3 Makerere University Biological Field Station, P.O. Box 967, Fort Portal, Uganda; 4 Nelson Institute for Environmental Studies, University of Wisconsin—Madison, Madison, WI, 53715, United States of America; 5 Department of Anthropology, McGill University, Montreal, Quebec, Canada, H3A 1B1; 6 Department of Biology, McGill University, Montreal, Quebec, Canada, H3A 1B1; 7 Department of Geography, McGill University, Montreal, Quebec, H3A 2T7, Canada; 8 Bilingual Biology Program, Department of Multidisciplinary Studies, Glendon Campus, York University, Toronto, Ontario, M3J 1P3, Canada; 9 Department of Pathobiological Sciences, University of Wisconsin-Madison, Madison, WI, 53706, United States of America; Centre for Cellular and Molecular Biology, INDIA

## Abstract

Substantial research has shown that while some parasite infections can be fatal to hosts, most infections are sub-clinical and non-lethal. Such sub-clinical infections can nonetheless have negative consequences for the long-term fitness of the host such as reducing juvenile growth and the host’s ability to compete for food and mates. With such effects, infected individuals are expected to exhibit behavioural changes. Here we use a parasite removal experiment to quantify how gastrointestinal parasite infections affect the behaviour of vervet monkeys (*Chlorocebus aethiops*) at Lake Nabugabo, Uganda. Behavioural profiles and the structure of nearest neighbour relationships varied significantly. As predicted, after deworming the duration of the resting events decreased, which is consistent with the idea that parasite infections are energetically costly. In contrast to what was predicted, we could not reject the null hypothesis and we observed no change in either the frequency or duration of grooming, but we found that the duration of travel events increased. A network analysis revealed that after deworming, individuals tended to have more nearest neighbours and hence probably more frequent interactions, with this effect being particularly marked for juveniles. The heightened response by juveniles may indicate that they are avoiding infected individuals more than other age classes because it is too costly to move energy away from growth. We consider that populations with high parasite burden may have difficulties developing social networks and behaviours that could have cascading effects that impact the population in general.

## Introduction

Earth’s ecosystems are increasingly disturbed by human activities [1] and infectious diseases are emerging at an increasing rate [2, 3]. These changes force people to cope with changing disease dynamics with regards to humanity and endangered species [4, 5]. Consequently, an understanding of how both the readily apparent (e.g., mortality[6]) and subtle (e.g., changes in behaviour [7]) effects of disease is becoming increasingly important. Severe parasitic infections can cause readily apparent symptoms including blood loss, tissue damage, spontaneous abortion, congenital malformations, and death [8–11]. Although substantial research has shown that while some parasite infections can be fatal to hosts, most infections are ordinarily sub-clinical and non-lethal [12, 13]. Such sub-clinical infections can nonetheless have negative consequences for the long-term fitness of the host such as reducing juvenile growth and the host’s ability to compete for food and mates. A well-documented case is found in cattle and sheep, where nematode infections can diminish weight gain, soft-tissue deposition, skeletal growth, and milk and wool production [14–17]. Similarly, Nilsson [18] showed that infected marsh tits were less reproductively successful as they experienced a rapid reduction in nestling growth. In general, parasite infections have energetic costs that affect the host’s resting metabolic rate (Robar et al. 2011, [19–21]). In white-footed mice (*Peromyscus leucopus*), for example, bot fly infection increases resting metabolic rate by 5% and the larva consumes 1% of the host’s nutrient budget [19]. In some cases, the cumulative effects of these factors can decrease the viability of the host population [22, 23]. For example, red grouse (*Lagopus lagopus*) with high levels of *Trichostrongylus tenuis* (a caecal nematode) were less fit as they encountered higher predation levels, thus limiting the population’s ability to grow [24].

With such effects, infected individuals are expected to exhibit behavioural changes, particularly given the increased metabolic rate and reduced energy and nutrient budgets associated with parasite infections [7]. For example, Ghai et al. [25] documented that whipworm (genus *Trichuris*) infection in red colobus monkeys (*Procolobus rufomitratus*) in Kibale National Park, Uganda was associated with behavioural shifts: monkeys decreased energetically costly behaviours when they were shedding whipworm eggs in faeces. The most commonly observed suite of behavioural responses to infection is termed “sickness behaviour” and encompasses reduced rates of movement and socialization, fatigue, lethargy, reduced food intake, and postures that reduce heat loss [7, 26, 27]. Previously, such sickness behaviours were considered maladaptive and a consequence of the parasite’s physiological demands on the host [28]. However, these behaviours are increasingly being interpreted as adaptive host responses to infections [7, 28, 29]. For example, a higher proportion of resting time and a lower proportion of feeding time have been documented in chimpanzees infected with an influenza-like virus [30]; these behavioural changes are likely part of a host strategy to combat infection. Another major activity of primates is grooming, which serves social functions and possibly removes ectoparasites [7, 31]. Grooming is a costly activity as it can create opportunity costs, energetic costs, and increased exposure to other parasites (e.g., pinworms [32–34]).

Our objective was to quantify how gastrointestinal parasite infections affect the behaviour of the vervet monkeys (*Chlorocebus aethiops*) at Lake Nabugabo, Uganda. We address this objective through a parasite removal experiment. Specifically, due to the energetic cost of parasite infection, we predicted that resting time would decrease and grooming time would increase after administering antiparasitic drugs. In addition, we assess possible relationships by examining how other behavioural change following the removal of gastrointestinal parasites. With these behaviours (e.g., duration of grooming, duration of resting, frequency of resting, node degree, node strength) we do not make specific predictions as understanding of causal relationships between changing behaviour and the effects of parasites is in its infancy and it is possible to logically derive multiple conflicting hypotheses [26]. This represents a continuation of our long-term research program that has previously documented behavioural changes in red colobus monkeys (*Procolobus rufomitratus*) in Kibale National Park, Uganda. Here, we found behavioural differences associated with gastrointestinal parasite infections, using a behavioural approach [25].

Our study was divided into four periods: **I)** Pre-study, where study subjects were habituated to the presence of researchers and we learned to individually recognize all group members, collected general activity data, and determined the population’s parasites, **II)** Phase 1, where behavioural and nearest-neighbour profiles were quantified and individual parasite infections were determined, **III)** Phase 2, where ivermectin was administered to eliminate each individual’s parasites, and **IV)** Phase 3, where post-“deworming” behaviour, nearest neighbour profiles, and parasite infections were quantified for comparison to Phase 1.

## Materials and Methods

### Study site and subjects

This research was carried out along the shores of Lake Nabugabo, Uganda (8.2 x 5 km), which is a satellite of Lake Victoria that lies at an elevation of 1,136 m (0°22’-12°S and 31°54’E), and the research station lies on the west side of the lake. The landscape around the lake is comprised of wetlands, grasslands, and patches of swamp forest, and the research site is comprised of farmer’s fields, degraded forest, and a few buildings. The area receives an average of 1,348 mm of rain annually. The north-south migration of the Intertropical Convergence Zone (ITCZ) is the primary influence on the annual distribution of rainfall, which results in a bi-modal rainfall pattern consisting of two rainy seasons (March through mid-May and November through early December) separated by two dry seasons (December through late February and mid-May through October) [35].

One group of vervet monkeys (M group), has been studied since June 2011. During the habituation period at the beginning of the study, dye-marking was employed to facilitate individual recognition; after this initial period, all individuals could be recognized by unique natural markings. Between May to August 2014, the study group consisted of 26 individuals (5 adult males, 8 adult females, 0 subadult males, 3 subadult females, and 10 juveniles and infants); however, only the adults and sub-adults are included in this experimental investigation (n = 16; with the exception of the nearest-neighbour analysis where all animals were included). Vervet monkeys are a useful model to examine the effects of changes in their parasite community as there have been a number of detailed studies of their parasites and the effect of these parasites have been described [36–38].

### Behavioural data collection

Scan samples and *ad libitum* data have been collected continuously since June 2011 and these were supplemented with focal animal samples during some phases of the study (see Study Design below). The study group was followed by either the investigators and/or trained field assistants for approximately 9 hours per day (7:00 to 16:00), 10 days a month in the pre-study phase and from dawn until dusk 6–7 days a week in Phases 1 through 3 (Phase 1 = 25 days, Phase 3 = 24 days). We collected scan data every 30-minutes on general activity, with detailed data on foraging. During these scans, we recorded the individual’s identity, sex, age category, behaviour (e.g., resting, moving [including canopy/ground level], feeding [including detailed information on food species, part, height from ground while foraging, and feeding rate whenever possible], self-grooming, social behaviour [i.e., giving or receiving grooming, playing]), nearest neighbour (in meters). In addition, in Phase 1 and 3 (before and after treatment), scans were used in the network analysis (see below). *Ad libitum* data were also collected on the group’s interactions with people, dogs, cattle, and crops (e.g., crop-raiding, foraging).

In Phases 1 and 3, we supplemented the scan and *ad libitum* behavioural data by conducted 15-minute focal animal sessions in which we continuously recorded the individual’s behaviour and nearest neighbour. All 16 adult and subadult study subjects were sampled daily, but the order in which they were sampled was on an *ad hoc* basis. No animal was resampled within the same 2 hours to ensure a fairly balanced sampling without spending a great deal of time locating individuals from a randomly generated list of study subjects. Focal data were used to construct individual activity budgets (% of observation time) that were considered as independent units in the analysis (see below). Data were discarded if focal individuals were out of sight for more than two minutes or more than one “out of sight” period occurred during the 15-minute focal observation. Moving was defined as a displacement of greater than two meters, primarily excluding short movements within a feeding patch.

### Detailed study design

#### Pre-study phase

Between June 2011 and April 2014, we monitored individual parasite infection and behaviour profiles on a monthly basis. This allowed us to determine which parasite species the group members hosted. These data allowed us to identify the likely route of transmission based on parasite life cycles, thereby enabling us to pay particular attention to behaviours that might influence parasite exposure risk in Phases 1 and 3. During the pre-study period, the study group was followed 10 days per month by either the investigators and/or trained field assistants for approximately 9 hours per day (7:00 to 16:00), during which we recorded 19,236 scans during 2,100 contact hours.

#### Phase 1

Between May 29 and June 2014, the “pre-deworming” phase, faecal samples for parasite analyses were collected intensively (average = 5.13, range = 3 to 10 per individual; details of the infections are reported in Valenta *et al*. (submitted)). Additionally, we determined behavioural patterns via scan sampling of all individuals, and focal animal sampling of all adult and subadults. The group was followed from dawn to dusk and 2,405 and 30- minute scans were conducted in 295 contact hours. We collected an average of 7.7 focal hours per individual (range per individual is 6.8 to 8.4)

#### Phase 2 (treatment)

Between June 24 and July 1, 2014, all adults and subadults were treated for helminth infections with two doses of ivermectin (0.3 mg/kg), with 5 days between dosages. A number of zoos and colonies regularly treat individuals for parasite infections and our methods are akin to those typically followed in captivity [39] and were deemed safe and supervised by two veterinarians (Dr. David Hyeroba (Ugandan Wildlife Vet) and Dr. Tony Goldberg (University of Wisconsin–Madison)). Ivermectin is often the anthelminthic of choice at a number of primate research centers because of its broad spectrum of activity against nematodes and safe application [40–42] and it has been used successfully on vervet monkeys [43]. With oral administration, the plasma half-life of ivermectin in humans is approximately 16 hours, thus after 3 days its concentration in the blood is minimal [40]. In this study, oral ivermectin was placed within a banana slice and given to specific individuals when they were foraging in isolation. This method of baiting had been well tested on the study group [44]. At this time, direct faecal smears of adults and subadults were carried out daily, with samples collected opportunistically. Treatment began on June 24^th^, 2014 and 15 of the 16 adults and subadults were successfully treated on this day. Two observers confidently viewed that two females (Ray and Limpy) spat out the ivermectin laced banana slices, so these individuals were given a second banana piece with ivermectin. The last individual was medicated on Wednesday, June 25^th^. On June 30^th^, the second dose was delivered to the 15 vervets who were originally medicated on 24^th^ and on July 1^st^ the last individual, who was treated one day late on the first treatment, received a second dose.

#### Phase 3

Between July 2–24, 2015, scan and focal animal sampling were resumed and faecal samples collected (average = 3.9, range = 2 to 7 per individual), to evaluate changes in behaviour that correspond to parasite removal. In this phase the group was again followed from dawn to dusk and 2705 scans were conducted in 249 contact hours. Focal animal sampling involved an average of 7.7 hours per individual in the pre-treatment sampling (range per individual is 6.8 to 8.4) and 8.2 hours post-treatment (range per individual is 7.2 to 9.2). Following the ivermectin treatment, no parasite eggs were found in faeces examined (average = 3.9, range = 2 to 7 per individual) via direct smear. It was during this 3 weeks that parasite re-infection from immediately infectious parasites with short prepatent periods (the interval between infection and the ability to diagnose infection, e.g., protozoans) was thought most likely to occur.

### Parasite evaluation

Fresh faecal samples were collected from all study individuals. Upon defecation, samples were immediately placed in vials and labelled by individual, date, location, and time of collection. At the end of the day, we weighed 1.0 g of wet faecal matter from each sample and stored it in 2.0 mL of 10% formalin solution for helminth parasite fixation. Sampling intensity varied during the different phases of the study (see Study Design below for details). In the three weeks prior to treatment (May 29^th^ to June 23^rd^, 2014), we collected five fecal samples from all adult and subadult members of M group for comparison to the three weeks after treatment (July 2^nd^ to July 24^th^), where again five samples were collected from each study animal to test efficacy of deworming treatment in the field. Parasite eggs were seen in the fecal matter of all tested animals before, but not after treatment.

Preserved fecal samples were examined for helminth eggs and larvae using a modified ethyl acetate sedimentation method, in which five slides of the sediment were examined for each sample [32, 45–47]. Thin preparations of sedimented feces were examined under 10X objective magnification on a Leica DM2500 light scope. Parasites were morphologically identified on the basis of egg color, shape, contents, and size, and photographed. Measurements were made to the nearest 0.1 mm ± SD using a calibrated ocular micrometer fitted to a compound microscope; parasites were photographed for further identification and documentation [32, 48]. Detailed species-level taxonomic identification of gastrointestinal parasites of wild primates from microscopic analysis alone is not possible, so we largely identified parasites to the genus level or higher.

Samples were considered positive for a particular parasite when one or more eggs or larvae were identified. The parasite infections were described in terms of richness and load (eggs/g). Richness is defined as the number of unique parasite species or types documented in each individual and an increase in richness could be indicative of greater morbidity. This is only an index of true richness as it is not possible to determine species-level identification based on egg morphology alone [46, 49].

### Data analysis

We used a paired t-test to examine behavioural changes before and after the ivermectin deworming treatment, with each of the 16 individuals serving as the independent unit of analysis. Specifically, we compared frequency of occurrence (percent of each activity based on the total number of events observed in the scan data) and the total duration (percent of total observation time for each individual calculated from the focal data) of each activity before and after anti-parasitic treatment using a paired t-test. These percentages were arc sign square-root transformed for analyses to ensure normality.

Data from scans were used to construct social networks in both the 3-weeks before deworming (Phase 1) and the 3-weeks after deworming (Phase 3). The nearest neighbour of each individual was used to create undirected social networks. Each node of the network denotes an individual, and edges connecting the different nodes indicates the presence of a nearest neighbor relationship between these nodes. For example, if *i* and *j* are two nodes in the network, then the presence of edge *ei*,*j* denotes that *i* and *j* were recorded as being nearest neighbours to one another at some point during the observation period. Each edge was then weighted with the frequency with which the pair of individuals were observed as nearest neighbours, such that the weighted edge, *w(ei*,*j)*, is the number of times *i* and *j* were recorded as being nearest neighbours. To account for likely biologically meaningless observations, edges with *w(ei*,*j)* = 1 were removed [50]; that is, we removed edges in which the two individuals were recorded as being nearest neighbours only once during the observation period—such episodes occurred when the group was travelling and individuals were spread out, thus we viewed the change of meaningful social interaction or interactions that could influence parasite transmission was low. Two nearest neighbour networks were created: one for the “pre-deworming” period (i.e., Phase 1) and one for the “post-deworming” period (i.e., Phase 3) [51]. This network analysis generated information on the frequency with which an individual associated with others, as well as the relative number of associations an individual had compared to others in the group. The number of individuals a node directly associates with is referred to as the node’s degree, while the sum of the weights of a node’s degree is called the strength of the node and captures the total number of associations a node has in the network [51]. Statistical contrasts were made among age/sex classes regarding their total number of associations (strength), with each individual being considered an independent point (i.e., node), using a one-way analysis of variance and subsequent Scheffe post hoc tests (identical conclusions were reached using non-parametric tests).

## Results

### Parasites

The most common infections included the following, Nematodes; *Ascaris* spp., which are generally considered benign infections in primates, though fatal cases have been reported in both Old World monkeys and great apes [52]; *Metastrongylus* spp., which are a lung worms that can cause haemorrhaging in the trachea, bronchi, and bronchioles [53]; *Oesophogostomum* spp. (nodule worm), infections characterized by adult worms in the bowel and of the juvenile stages enclosed in “nodules” in the intestinal wall, the liver, and the abdominal wall [52, 54–56]; *Strongyloides* spp. (likely *S*. *fuelleborni*), which commonly infect primates, and autoinfection can occur [57–59]; *Toxocara spp*., which are generally benign infections; *Trichostrongylus* spp., whose occurrence has not been documented in any other vervet population [32]); *Trichuris* spp., or whipworms, are generally benign, though since adults produce 2,000–10,000 eggs/day and since infections often involve many adults [45, 53], they can be an energy burden. A recent molecular study found that the host of *Trichuris* varied by taxonomic group with some whipworms being host specific, while others were generalist in the primate community of Kibale National Park, Uganda. This vervet population also hosts to a number of trematodes, including *Fasciola* spp. (liver flukes), which among wild vervet monkey populations have only been described in the vervets of Nabugabo. This population may be more likely to contact the infective stage metacercariae than animals in other populations as they forage along the lake shore, where they are safe from predatory dogs (see also [60]) (for a complete list and description see [32]).

No parasite eggs were found in the first month after the ivermectin treatment. Four months after treatment, cestode eggs (*Strongyloides* spp.) and trematode eggs (*Fasciola* spp.) were found in a number of faecal samples, indicating study subjects had become re-infected. Given published prepatent periods, we suspect that infective stages were ingested during Phase 3 of the study.

### Behaviour before and after anthelminthic treatment (Phase 1 vs Phase 3)

Individuals rested more frequently after treatment (44%) than before treatment (25%; paired t = 2.281, p = 0.038). Although the number of resting bouts increased after treatment, the duration of resting decreased by 75.8% after the treatment (paired t = 2.851, p = 0.013). Additionally, the duration of travel events increased by 53.4% after treatment (paired t = 4.041, p = 0.001). No other behavioural comparisons differed between the “pre-deworming” period and the “post-deworming” period (all p-values were p >0.17).

### Network analysis

**Fig 1:**
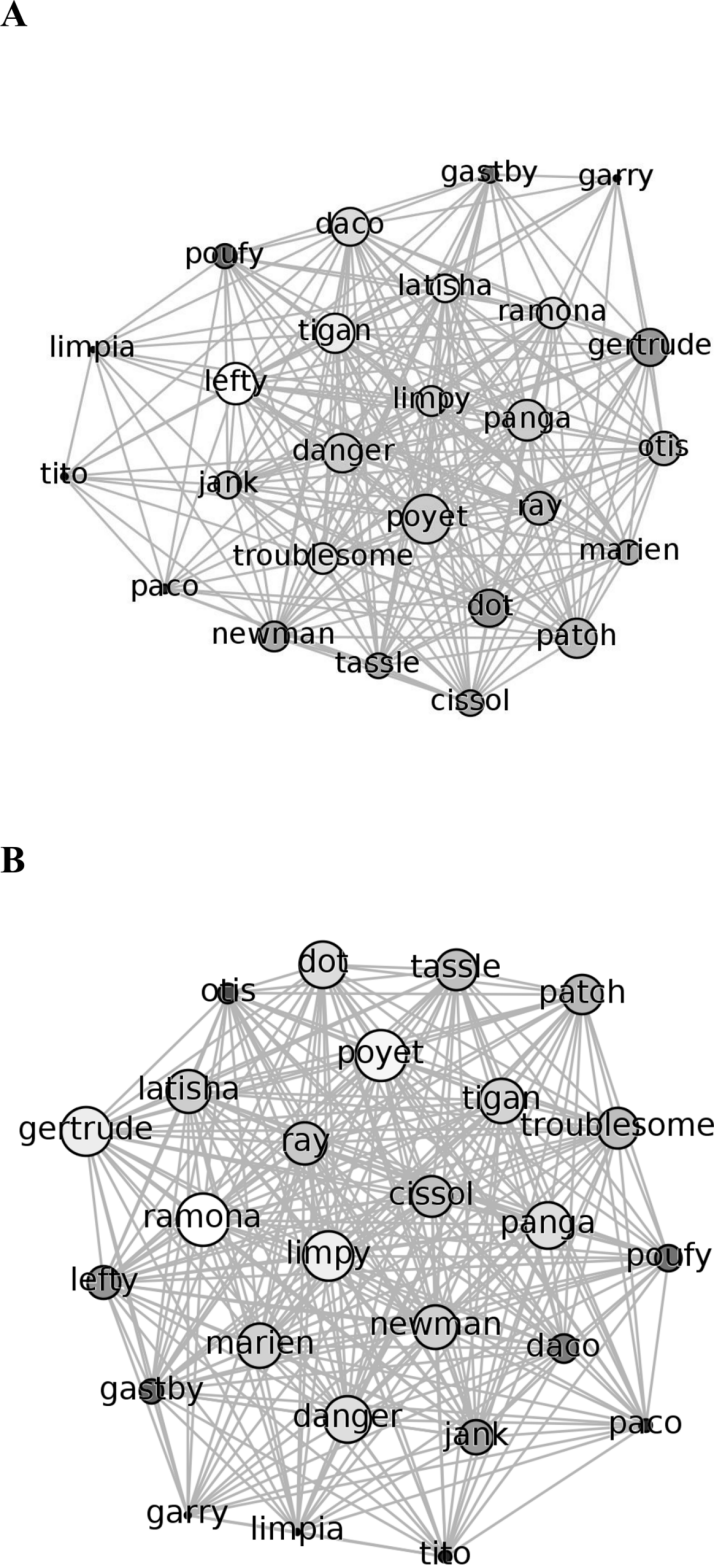
**The nearest neighbour network of the primate population (A) before and (B) after de-worming**. The layout of the nodes and edges were determined by using the ForceAtlas2 algorithm [61] with identical parameterization. The well-connected nodes occupy central positions in the graph. The size of the nodes are scaled to the sum of the weights of all edges incident upon it. The nodes are also shaded such that nodes with a higher degree are lighter in color.

Two nearest neighbour networks were created based on 1,864 observations before deworming and 2,404 observations after (nearest-neighbour distance was not possible to collect in all scans). Since the length of the observation periods were the same both before and after deworming (three weeks) and the number of hours were similar (122 versus 131 hours), the increase in the number of observations can likely be primarily attributed to the increase in the duration of travel events, as the increased movement caused the nearest neighbour to change more often than when the individuals were resting or feeding. Despite the larger number of observations in the latter network, the general topology of each network appeared very similar with respect to the nature of associations before and after the deworming (Fig 1A and 1B). However, close inspection of the individual nodes revealed interesting changes in the nearest neighbour networks before and after deworming for some individuals. Prior to deworming, juveniles 1 to 3 (named Garry, Tito and Limpia) were on the fringes of the network as a result of their lack of associations, but increased associations after deworming led to a move closer to the centre of the network. Even though almost all the individuals recorded higher associations, the relative increase to the three juveniles was large enough to draw them closer to the core of the network using the same algorithm and parameterization for rendering. Fig 1A and 1B show the weighted networks, in which the size of the individual nodes is scaled according to their degree of associations with other nodes. The increase in the size of nodes representing these juveniles, highlights the increase in the number of individuals they had as nearest neighbour after de-worming.

Analyses of the number of individuals each animal interacted with and the frequency of associations (Table 1) reveal that both increased after deworming (mean number of individuals before = 19.5, after = 22.5, paired t-test = 5.043, P<0.001, paired by individual; mean frequency of associations before = 127.2, after 210.5, paired t-test = 11.832, P<0.001). Furthermore, analysis of variance supports the visual inspection of the network (Fig 1A and 1B). The difference in degree of centrality indicating that with respect to the number of individuals an animal associated with, the difference between before and after deworming was greater for juveniles than adult females and subadult females (F = 5.45, P = 0.004). With respect to the frequency of associations, juveniles before and after deworming were greater for juveniles than adult females and females with an infant (F = 6.10, P = 0.002, Scheffe post-hoc analysis).

**Table 1:** The number of individuals each individual interacts with (Degree) as well as the total number of associations (strength) that were observed during the period before and after deworming.

Age/Sex	Individual	Before		After	
		*Degree*	*Total number of associations (strength)*	*Degree*	*Total number of associations (strength)*
Juvenile 1	Garry	10	49	18	91
Juvenile 2	Tito	10	54	19	95
Juvenile 3	Limpia	11	54	19	117
Juvenile 4	Paco	12	62	19	118
Juvenile 5	Gastby	16	85	23	164
Juvenile 6	Poufy	16	111	20	174
Adult Female 1	Dot	19	113	22	245
Adult Female 2	Gertrude	19	115	21	232
Adult Male 1	Cissol	20	118	25	232
Female+Infant 1	Marien	20	120	25	206
Adult Male 2	Newman	20	125	25	242
Adult Female 3	Tassle	20	131	22	267
Adult Male 3	Otis	21	133	21	243
Adult Female 5	Patch	21	133	20	231
Adult Female 6	Ray	21	134	24	278
Subadult Female 1	Danger	22	141	25	238
Adult Male 4	Jank	22	143	25	146
Adult Female 7	Limpy	22	156	25	265
Subadult Female 2	Panga	22	157	24	253
Adult Male 5	Poyet	22	158	23	182
Juvenile 7	Daco	23	161	24	254
Female+infant 2	Ramona	23	162	24	226
Adult Female 8	Troublesome	23	163	23	246
Adult Female 9	Latisha	24	168	23	254
Subadult Female 3	Tigan	24	169	24	201
Subadult Male 1	Lefty	25	193	23	272

## Discussion

We investigated changes in host behaviour and nearest neighbour relationships in relation to gastrointestinal infections before and after a deworming procedure. Individual behavioural profiles and the structure of their nearest neighbour relationships varied significantly, but subtly in a fashion that could be of important biological significance, particularly for juveniles. As predicted, after deworming the duration of the resting events decreased [7, 28, 29], which is consistent with the idea that parasite infections are an energetic cost to hosts [62]. However, individuals rested more frequently; the reason for this is not clear. A higher proportion of resting time and a lower proportion of feeding time have been documented in chimpanzees infected with an influenza-like virus [30].

In contrast to what was predicted, In contrast to what was predicted, we could not reject the null hypothesis that there was no change in either the frequency or duration of grooming, but we found that the duration of travel events increased. This increase in the duration of traveling may reflect the fact that parasite infections have been removed and thus animals have more energy and spend more time travelling to the best/most nutritious feeding sites. To verify this speculation, it would be interesting to examine the feeding rate and nutritional quality of the foods eaten before and after deworming.

The network analysis revealed that after deworming, individuals tended to have more nearest neighbours and more frequent interactions. Of particular interest is the fact that juveniles exhibited the greatest response. Juveniles are the age class that is arguably most affected by levels of interaction because of their importance in socialization and learning [63, 64]. We speculate that it is possible that this heightened response by juveniles indicates that they are avoiding infected individuals more than other age classes because it is too costly to have a parasite infection that would move energy away from growth; thus they invest less energy into social interactions (mechanisms that vervet monkeys evaluate the infection level of other individuals must be confirmed for this speculation to be possible). Alternatively, the shift could be a function of the energy the juveniles have after travel costs are met. Such a shift in energy allocation could have important impacts on the individual that could have cascading effects. In fact, such changes in individual behavioural patterns may impact the population in general, which could have serious consequences particularly for endangered species. Our findings leads us to speculate that populations experiencing high parasite burden will have reduced foraging abilities as learning what are appropriate food sources (the types of food that are most nutritious) will be poor. This learning would be inhibited because the rate of interactions would be lowered by the infection as indicated here for the juveniles. In addition, such populations with high parasite burden may have difficulties developing social networks and behaviours that may facilitate reproductive success. Both of these changes that potentially result from increased disease may decrease the reproductive potential of individuals and populations [26, 65]. Disease may thus be a route that decreases population growth or recovery after disturbance, and thereby influences the population’s conservation potential [66, 67]. Such speculations need to be validated, but point to interesting avenues for future research that unify studies of parasites, behaviour, and conservation.

## References

[1] Hansen MC, Potapov PV, Moore R, Hancher M, Turubanova SA, Tyukavina A, et al High-resolution global maps of 21st-century forest cover change. Science. 2013;342:850–3. 10.1126/Science.1244693 24233722

[2] Jones KE, Patel NG, Levy MA, Storeygard A, Balk D, Gittleman JL, et al Global trends in emerging infectious diseases. Nature. 2008;451:990–4. 10.1038/nature06536 . 18288193 PMC5960580

[3] Nunn CL, Thrall PH, Stewart K, Harcourt AH. Emerging infectious diseases and animal social systems. Evol Ecol. 2008;22:519–43. 10.1007/s10682-007-9180-x .

[4] Goldberg TL, Chapman CA, Cameron K, Saj S, Karesh W, Wolfe N, et al Serologic evidence for a novel poxvirus in endangered red colobus monkeys. Emerging Infect Dis. 2008;14:801–3. 10.3201/eid1405.071686 18439366 PMC2600227

[5] Walsh PD, Abernethy KA, Bermejo M, Beyersk R, De Wachter P, Akou ME, et al Catastrophic ape decline in western equatorial Africa. Nature. 2003;422:611–4. 10.1038/nature01566 . 12679788

[6] Young TP. Natural die-offs of large mammals: implications for conservation. Conserv Biol. 1994;8:410–8.

[7] Hart BL. Behavioural adaptations to pathogens and parasites—5 strategies. Neuroscience and Biobehavioral Reviews. 1990;14(3):273–94. . 2234607 10.1016/s0149-7634(05)80038-7

[8] Collias N, Southwick C. A field study of population density and social organization in howling monkeys. Proc Natl Acad Sci. 1952;96:143–56.

[9] Despommier DD, Gwazda RW, Hotez PJ. Parasitic Diseases. New York: Springer-Verlag; 1995.

[10] Ayuda MI, Pinilla V. El proceso de desertización demográfica en la montaña Pirenaica el largo plazo: Aragón. Ager: Revista de Estudios de Despoblación y Desarrollo Rural. 2002;2:101–38.

[11] Hudson PJ, Rissoli A, Grenfell BT, Heesterbeek H, Dobson AP, editors. The ecology of wildlife diseases Oxford: Oxford University Press; 2001.

[12] Munger JC, Karasov WH. Subleathal parasites and host energy budgets: Tapeworm infection in white-footed mice. Ecology. 1989;70:904–21.

[13] Murray DL, Cary JR, Keith LB. Interactive effects of subleathal nematodes and nutritional status on snowshoe hare vulnerability to predation. J Anim Ecol. 1997;66:250–64.

[14] Parkins JJ, Holmes PH. Effects of gastrointestinal helmint parasites on ruminant nutrition. Nutrition Research Reviews. 1989;2:227–46. 10.1079/NRR19890016 19094355

[15] Poppi DP, Sykes AR, Dynes RA. The effect of endoparasitism on host nutritioon–the implications for nutrient manipulation. Proceedings of the New Zealand Society of Animal Production. 1990;50:237–43.

[16] Holmes PH. Interactions between parasites and animal nutrition: the veterinary consequences. Proceedings of the Nutrition Society. 1993;52:113–20. 8493256 10.1079/pns19930043

[17] Sykes AR. Parasitism and production in farm animals. Anim Prod. 1994;59:155–72.

[18] Nilsson JA. Ectoparasitism in marsh tits: costs and functional explanations. Behavioural Ecology. 2003;14:175–81.

[19] Munger JC, Karasov WH. Costs of bot-fly infection n white-footed mice: Energy and mass flow. Canadian Journal of Zoology. 1994;72:166–76.

[20] Robar N, Murray DL, Burness G. Effects of parasites on host energy expenditure: the resting metabolic rate stalemate. Can J Zool/Rev Can Zool. 2011;89:1146–55.

[21] Hechinger RF. A metabolic and body-size scaling framework for parasite within-host abundance, biomass and energy flux. Am Nat. 2013;182:234–48. 10.1086/670820 23852357

[22] Ewald PW. Host-parasite relations, vectors and the evolution of disease severity. Annu Rev Ecol Syst. 1983;14:465–85.

[23] Schmid-Hempel P, DEbert D. On the evolutionary ecology of specific immune defence. Trends Ecol Evol. 2003;18:27–32.

[24] Hudson PJ, Dobson AP, Newborn D. Do parasites make prey vulnerable to predation: red grouse and parasites. J Anim Ecol. 1992;61:681–92.

[25] Ghai RR, Fugere V, Chapman CA, Goldberg TL, Davies TJ. Sickness behaviour associated with a non-lethal parasite in wild primate. Proceedings of the Royal Society of London, Biology. in press. 10.1098/rspb.2015.1436 PMC4571704 26311670

[26] Nunn CL, Altizer S. Infectious diseases in primates: Behavior, ecology and evolution Oxford: Oxford University Press, Oxfor, U.K.; 2006.

[27] Beros S, Jongepier E, Hagemeier F, Foitzik S. The parasite's long arm: a tapeworm parasite induces behavioural changes in uninfected group members of its social host. Proceedings of the Royal Society of London, Biology. 2015;282:20151473. 10.1098/rspb.2015.1473 PMC4685803 26582019

[28] Poulin R. “Adaptive” changes in the behaviour of parasitized animals: a critical review. International Journal of Parasitology. 1995;25:1371–83. 8719948 10.1016/0020-7519(95)00100-x

[29] Hart BL. Biological basis of the behavior of sick animals. Neurosci Biobehav Rev. 1988;12:123–37. 3050629 10.1016/s0149-7634(88)80004-6

[30] Krief S, Hladik CM, Haxaire C. Ethnomedicinal and bioactive properties of plants ingested by wild chimpanzees in Uganda. J Ethnopharmacol. 2005;101:1–15. 15913933 10.1016/j.jep.2005.03.024

[31] Kynkaanniemi SM, Kettu M, Kortet R, Harkonen L, Kaitala A, Paakkonen T, et al Acute impacts of the deer ked (Lipoptena cervi) infestation on reindeer (*Rangifer tarandus tarandus*) behaviour. Parasitol Res. 2014;133:1489–97. 10.1007/s00436-014-3790-3 24562815

[32] Valenta K, Twinomugisha D, Liu C, Schoof VA, Goldberg TL, Chapman CA. Habitat effects on gastrointestinal parasites in vervet monkeys (*Chlorocebus aethiops*). Integrative Zoology. Submitted. 10.1111/1749-4877.12270 PMC5725676 28685946

[33] Tutin CEG. Ecology and social organisation of African rainforest primates: relevance for understanding the transmission of retroviruses. Bulletin De La Societe De Pathologie Exotique. 2000;93(3):157–61. . 11030048

[34] Hasegawa H. Methods of collection and identification of minute nematodes from the feces of primates, with special application to coevolutionary study of pinworms In: Huffman MA, Chapman CA, editors. Primate Parasite Ecology: The Dynamics and Study of Host-Parasite Relationships. Hasegawa H. Huffman M.A. and Chapman C.A. editors. Cambridge Studies in Biological and Evolutionary Anthropology, Cambridge University Press, Cambridge. pp, 487–506. Cambfidge: Cambridge University Press; 2009 p. 29–46.

[35] Stampone M, Hartter J, Chapman CA, Ryan SJ. Trends and variability in localized percipitation around Kibale National Park, Western Uganda, Africa. Research Journal of Environmental and Earth Sciences. 2011;3:14–23.

[36] Wren BT. Gastrointestinal parasites of the vervet monkey (*Chlorocebus [Cercopithecus] aethiops*) at a Sanctuary in Limpopo Province, South Africa. Program for 122nd Annual Meeting of the Indiana Academy of Science. 2006;1:42.

[37] Wren BT, Gillespie TR, Camp JW, Remis MJ. Helminths of Vervet Monkeys, Chlorocebus aethiops, from Loskop Dam Nature Reserve, South Africa. Comp Parasitol. 2015;82(1):101–8.

[38] Gaetano TJ, Danzy J, Mtshali MS, Theron N, Schmitt CA, Grobler JP, et al Mapping correlates of parasitism in wild South African vervet monkeys (Chlorocebus aethiops). S Afr J Wildl Res. 2014;44(1):56–70.

[39] Johnson-Delaney CA. Parasites of captive nonhuman primates. The veterinary clinics of North America. Exotic Animal Practice. 2009;12:563–81. 10.1016/j.cvex.2009.07.002 19732709

[40] Dufour JP, Cogswell FB, Phillippi-Falkenstein KM, Bohn RP. Comparison of efficacy of moxidectin and ivermectin in the treatment of *Strongyloides fulleborni* infection in rhesus macaques. Journal of Medical Primatology. 2006;35:172–6. 16764676 10.1111/j.1600-0684.2006.00154.x

[41] Wang T, Yang G-Y, Yan H-J, Wang S, Bian Y, Chen A-C, et al Comparison of efficacy of selamectin, ivermectin and mebendazole for the control of gastrointestinal nematodes in rhesus macaques, China. Vet Parasitol. 2008;153:121–5. 10.1016/j.vetpar.2008.01.012 18295404

[42] Mati VLT, Junior FCF, Pinto HA, de Melo AL. *Strongyloides cebus* (Nematoda: Strongyloididae) in *Lagothrica cana* (Primates: Atelidae) from the Brazilian Amazon: Aspects of clinical presentation, anatomopathology, treatment, and parastic biology. J Parasitol. 2013;99:1009–18. 10.1645/13-288.1 23909511

[43] Mukaratirwa S, Dzoma BM, Matenga E, Ruziwa SD, Sacchi L, Pozio E. Experimental infection of baboons (*Papio* spp.) and vervet monkeys (*Cercopithecus aethiops*) with *Trichinella zimbabwensis* and successful treatment with ivermectin. Onderstepoort J Vet Res. 2008;75:173–80. 18788211

[44] Teichroeb JA, Chapman CA. Sensory information and associative cures used in food detection by wild vervet monkeys. Animal Cognition. 2014;17:517–28. 10.1007/s10071-013-0683-2 24045849

[45] Garcia LS. Practical guide to diagnostic parasitology Washington, D.C.: ASM Press; 1999.

[46] Greiner EC, McIntosh A. Collection methods and diagnostic procedures for primate parasitology In: Huffman MA, Chapman CA, editors. Primate parasite ecology: the dynamics and study of host-parasite relationships. Cambridge: Cambridge University Press; 2009 p. 3–28.

[47] Sloss MW, Kemp RL, Zajac AM. Veterinary Clinical Parasitology. 6 ed. Ames: Iowa State University Press, Iowa; 1994.

[48] Chapman CA, Wasserman MD, Gillespie TR, Speirs ML, Lawes MJ, Saj TL, et al Do nutrition, parasitism, and stress have synergistic effects on red colobus populations living in forest fragments? Am J Phys Anthropol. 2006;131:525–34. 16958077 10.1002/ajpa.20477

[49] Ghai RR, Simons ND, Chapman CA, Omeja PA, Davies TJ, Ting N, et al Hidden population structure and cross-species transmission of whipworms (*Trichuris* sp.) in humans and non-human primates in Uganda. PLoS Neglected Tropical Diseases. 2014;8:e3256 10.1371/journal.pntd.0003256 25340752 PMC4207677

[50] James R, Croft DP, Krause J. Potential banana skins in animal social network analysis. Behav Ecol Sociobiol. 2009;63:989–97.

[51] Sueur C, Amblard F, Petit O, King AJ. How can social network analysis improve the study of primate behavior? Am J Primatol. 2011;78:703–19. 10.1002/ajp.20915 21181869

[52] Gillespie TR, Greiner EC, Chapman CA. Gastrointestinal parasites of the colobus monkeys of Uganda. J Parasitol. 2005;91:569–73. . 16108549 10.1645/GE-434R

[53] Cheng TC. General Parisitology. New York: Academic Press; 1973.

[54] Gasser RB, de Gruijter JM, Polderman AM. Insights into the epidemiology and genetic make-up of *Oesophagostomum bifurcum* from human and non-human primates using molecular tools. Parasitology. 2006;132:453–60. 16332292 10.1017/S0031182005009406

[55] Polderman AM, Blotkamp J. *Oesophagostomum* infections in humans. Parasitol Today. 1995;11:451–6. 15275382 10.1016/0169-4758(95)80058-1

[56] Ghai RR, Chapman CA, Omeja PA, Davies TJ, Goldberg TL. Nodule worm infection in humans and wild primates in Uganda: cryptic species in a newly identified region of human transmission. PLoS Neglected Tropical Diseases. 2014;8:e2641 10.1371/journal.pntd.0002641 24421915 PMC3888470

[57] Sa RM, Petrasova J, Pomajbikova K, Profousová I, Petrželková KJ, Sousa C, et al Gastrointestinal symbionts of chimpanzee in Cantanhez National Park guinea-bissau with respect to habitat fragmentation. Am J Primatol. 2013;75:1032–41. 10.1002/ajp.22170 23776090

[58] Mati VLT, Ferreira FC, Pinto HA, de Melo AL. *Strongyloides cebus* (Nematoda: Strongyloididae) in Lagothrix cana (Primates: Atelidae) from the Brazilian Amazon: Aspects of clinica presentation, anatomopathology, treatment and parasitic biology. J Parasitol. 2013;99:1009–18. 10.1645/13-288.1 23909511

[59] Bowman DD. Georgis' parasitology for veterinarians. 7th ed. St Louis: Elsevier; 1999.

[60] James C, Adobison AR, Harrison RA, Nelson GS. Long-term infection of S*chistosma mansoni* in a veret monkey (*Cercopithecus aethiops*). Trans R Soc Trop Med Hyg. 1983;77:51–2. 6679365 10.1016/0035-9203(83)90012-3

[61] Jacomy M, Venturini T, Heymann S, Bastian M. ForceAtlas2, a continuous graph layout algorithm for handy network visualization designed for the gephi software. PLOS One. 2014;9:e98679 10.1371/journal.pone.0098679 24914678 PMC4051631

[62] Sheldon BC, Verhulst S. Ecological immunology: Costly parasite defences and trade-offs in evolutionary ecology. Trends Ecol Evol. 1996;11(8):317–21. . 21237861 10.1016/0169-5347(96)10039-2

[63] Deputte BL. Primate socialization revisited: theoretical and practical issues in social ontogeny. Advances in the Study of Behavior. 2000;29:99–157.

[64] Glander KE. The impact of plant secondary compounds on primate feeding behavior. Yearbook of Physical Anthropology 1982;25:1–18.

[65] Chapman CA, Gillespie TR, Goldberg TL. Primates and the ecology of their infectious diseases: How will anthropogenic change affect host-parasite interactions? Evolutionary Anthropology. 2005;14:134–44. .

[66] Altizer S, Nunn CL, Lindenfors P. Do threatened hosts have fewer parasites? A comparative study in primates. J Anim Ecol. 2007;76(2):304–14. 10.1111/j.1365-2656.2007.01214.x . 17302838

[67] Pedersen AB, Jones KE, Nunn CL, Altizer S. Infectious diseases and extinction risk in wild mammals. Conserv Biol. 2007;21(5):1269–79. 10.1111/j.1523-1739.2007.00776.x . 17883492 PMC7202242

